# Supplementary material for: High throughput intracellular delivery by viscoelastic mechanoporation
Source: Nat Commun. 2024 Jan 2;15:115. doi: 10.1038/s41467-023-44447-w (PMC10762167; doi:10.1038/s41467-023-44447-w)
Supplement: Supplementary file 1 — Supplementary Information [file 41467_2023_44447_MOESM1_ESM.pdf]

Supplementary Information

High throughput intracellular delivery by viscoelastic mechanoporation

Derin Sevenler and Mehmet Toner

Supplementary Note 1: Dimensionless numbers and their approximate values for viscoelastic mechanoporation

Reynolds Number	$Re = \frac{\rho \bar{U} d}{\eta} \approx 62.5$	Ratio of fluid inertia to viscous stresses
Weissenberg Number	$Wi = \lambda \frac{\bar{U}}{d} \approx 6{,}250$	Ratio of elastic stresses to viscous stresses
Elasticity Number	$El = \frac{Wi}{Re} = \frac{\lambda \eta}{\rho d^2} \approx 100$	Ratio of elastic stresses to fluid inertia

The Reynolds number, Weissenberg number, and Elasticity number describe the relative magnitudes of fluid inertia, viscous stress, and elastic stress within the microfluidic contraction at the narrowest point. Note that the Elasticity number is defined as the ratio of the Weissenberg Number to the Reynolds Number and does not depend on flow rate. Fluid and geometric parameters used in these definitions are listed here:

Deformation length scale	$d = \frac{w}{2} \approx 25\ \mu m$
Flow speed at the contraction	$\bar{U} = \frac{Q}{wh} \approx \frac{3\ \text{mL/min}}{(50\ \mu\text{m})(80\ \mu\text{m})} = 12.5\ \text{m/sec}$
Polymeric relaxation time	$\lambda \approx 12.5\ \text{msec}$
Characteristic zero-shear viscosity	$\eta_0 \approx 5\ \text{mPa} \cdot \text{sec}$<br>Solvent contribution: $\eta_s \approx 1\ \text{mPa} \cdot \text{sec}$<br>Polymeric contribution: $\eta_p \approx 4\ \text{mPa} \cdot \text{sec}$
Density	$\rho \approx 1000 \frac{kg}{m^3}$

The values of these numbers for the contraction are roughly approximated based on the flow rate, geometry (channel width w and height h), and rheological values for 1 mg/mL 1.6 MDa hyaluronic acid in PBS based on measurements in the available literature.[1–3]

Note the definition used above for the Weissenberg number is based on laminar channel flow (Poiseuille flow), however in both simulations and experiments the flow within the channel is not fully developed. Care should be taken when making direct comparisons with other reports based on Wi alone.

Supplementary Note 2: Onset of contraction vortices and the viscoelastic Mach number

To contextualize the experimental and computational studies of the upstream vortices, we briefly consider the viscoelastic Mach number, $Ma_{el} = \frac{\bar{U}}{c}$, a dimensionless number comparing the mean flow velocity $\bar{U}$ to the elastic wave speed $c = \sqrt{\frac{\eta_p}{\rho\lambda}}$.[4] The transition to $Ma_{el} > 1$ has previously been identified as relevant to the onset of flow separation for contracting flows in the inertio-elastic regime (i.e., where both Re and Wi are large).[4,5] Indeed, the onset and growth of upstream separated flow in the computational results was observed to coincide with regions where the local Ma_{el} exceeded one in the upstream region (**Figure S8**).

Supplementary Note 3: A large contraction ratio is required for strain hardening

The extensional viscosity of a polymer solution tends to increase with both the deformation rate ("tension thickening") and deformation timescale ("strain hardening") as faster and larger deformations create a disproportionately greater degree of extension and alignment in the polymer chains.[6] In this work, the channel contraction was made as large as practical to maximize strain hardening, making it possible to achieve sufficient membrane tensions for mechanoporation with a dilute polymer solution. In the final microfluidic device, the extensional Hencky strain of the contraction was $\epsilon_H = \ln(CR) = \ln\left(\frac{1{,}500\ \mu m \cdot 80\ \mu m}{50\ \mu m \cdot 80\ \mu m}\right) \approx 3.4$, where CR is the channel contraction ratio (i.e., the ratio of the upstream channel cross sectional area to the area at the narrowest point). Small stretches (i.e., Hencky strains less than approximately 2), even if performed quickly, are typically not sufficient to "turn on" the polymeric contribution to the extensional viscosity.[7,8] These considerations are important for contrasting this work with other recent studies using nonlinear fluid forces for mechanoporation.[9]

Supplementary Table 1: Comparison of viscoelastic mechanoporation to existing and emerging membrane disruption technologies.

[9–20]

Technology	Reference	**Characteristic yield** (Efficiency x Viability)	**Cell throughput** (millions per minute)	**Cell concentration** (millions per mL)
Viscoelastic mechanoporation	This work	90%	250	100
µ-cell stretcher	Kwong et al, 2023[9]	50%	0.35	1
µF cell stretching	Hur et al, 2020[10]	75%	1	1
iMCH	Kizer et al, 2019[11]	80%	1.6	1
iMCH	Deng et al, 2018[12]	40%	1	1
Spiral hydroporator	Kang et al, 2020[13]	60%	1	2
µVS	Jarrell et al, 2021[14]	30%	60	15
µVS	Jarrell et al, 2019[15]	40%	120	16
SQZ-EP	Ding et al, 2017[16]	30%	5	3
µEP	Lissandrello et al, 2020[17]	80%	20	50
VECT	Liu et al, 2018[18]	80%	5	5
DMP	Dixit et al, 2020[19]	80%	0.01	Not stated
ASP	Meacham et al, 2018[20]	80%	6	1

Supplementary Table 2. crRNA sequence selected for TCR knockout study. crRNA was synthesized by Integrated DNA technologies and included both 5’ and 3’ DT Alt-R® end-blocking modifications.

**crRNA sequence**	AG AGU CUC UCA GCU GGU ACA GUU UUA GAG CUA UGC U

Supplementary Table 3: Reagent cost breakdown

Relative costs of buffer components and reagents per 200 μL reaction (e.g., 1-20 million cells):

Component	Nominal cost per 200 μL, USD	Vendor
0.5 mg/mL Hyaluronic acid, 1.6 MDa	**$0.06** (0.4 mg x $0.15/mg)	Lifecore Biomedical
1x Phosphate buffered saline	**$0.007** (200 μL x $38/L)	Sigma-Aldrich
100 μg/mL eGFP mRNA	**$80** (20 μg x $4/μg)	Fisher Scientific
1 μM Cas9 protein	**$12** (8 μg x $1.50/μg)	Integrated DNA Technologies

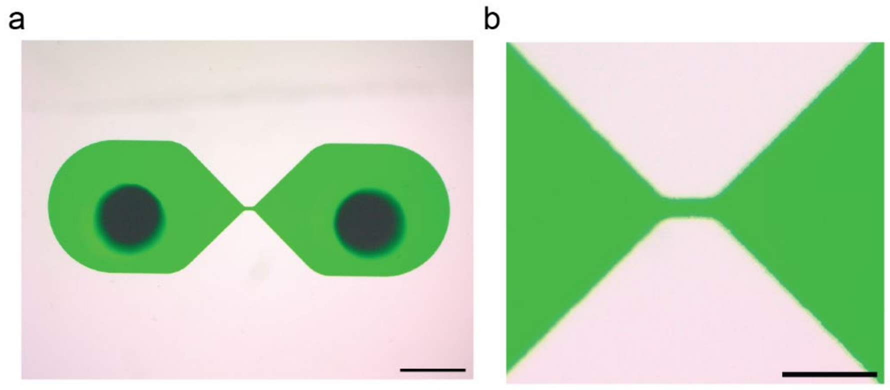

Supplementary Figure 1. Images of initial microfluidic device without upstream cell focusing, visualized with green dye. Scalebars indicate (a) 1 mm and (b) 250 µm. Images of replicate devices were similar.

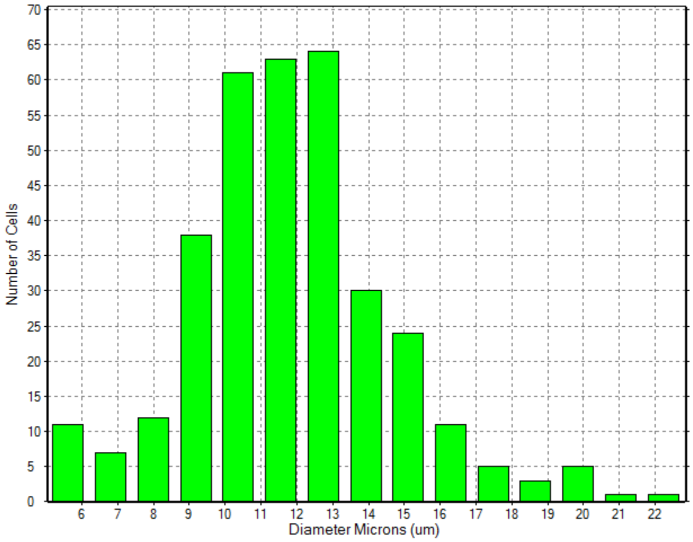

Supplementary Figure 2. Histogram of Jurkat cell size (Cellometer, Nexcelom Biosciences).

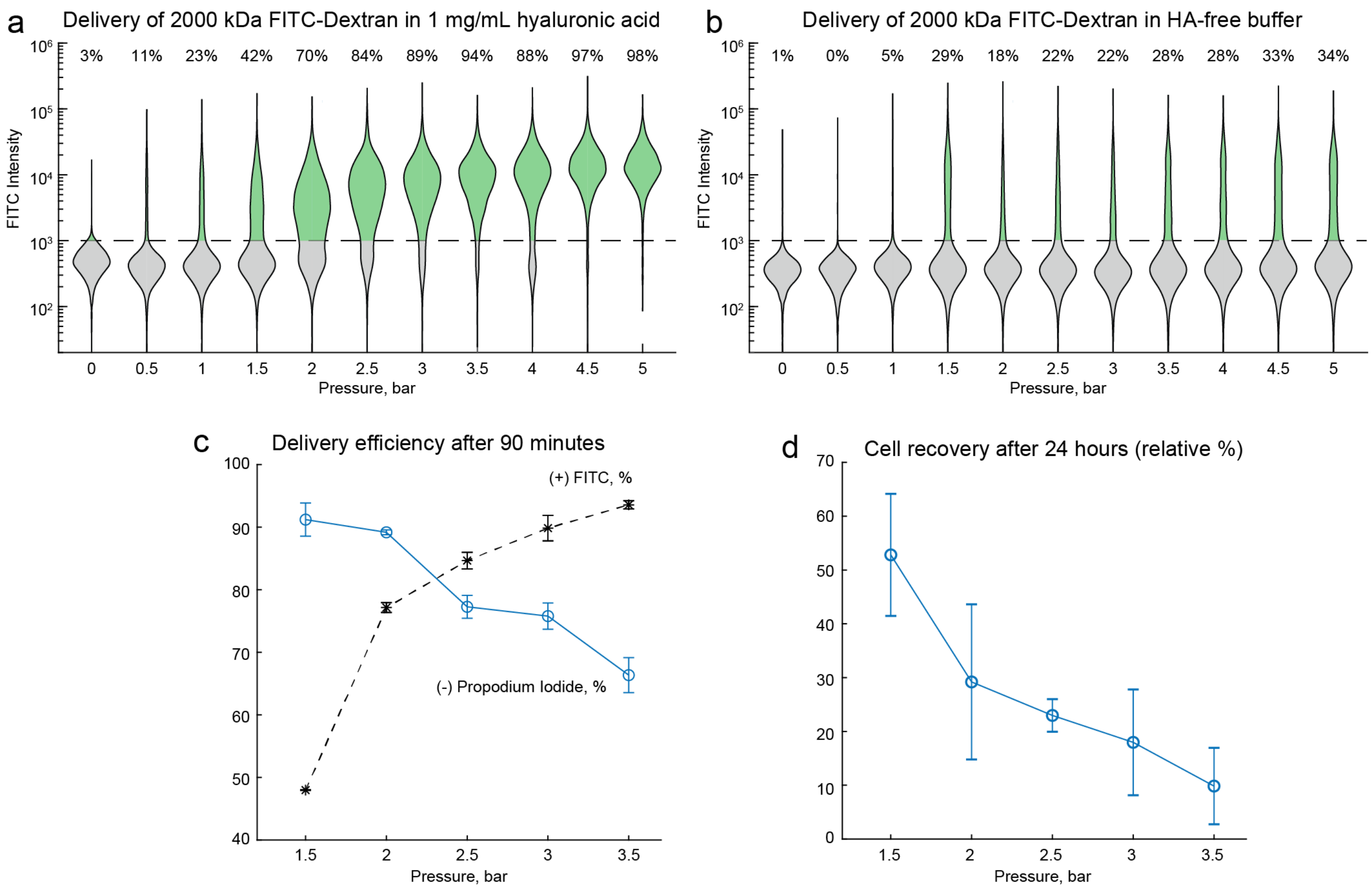

Supplementary Figure 3. Transfection performance in the contraction-only device. Distributions of FITC intensities for viable cells after processing in the channel-only device (Supplementary Figure 1) at increasing driving pressure with a transfection solution containing (a) 1 mg/mL hyaluronic acid (HA) or (b) no HA. A pressure of '0 bar' on the x-axis is used for samples which were incubated with the transfection buffer but not processed through the microfluidic chip. Percentages above each distribution indicates delivery efficiency, i.e., percentage of cells above the threshold denoted by the dashed line. (c) Delivery efficiency and viability by propidium iodide exclusion, 90 minutes after stretching in 1 mg/mL HA ($n = 3$). (d) Total cell recovery relative to unstretched controls following overnight culture ($n = 3$). Error bars in (c, d) indicate ± one standard deviation of the mean. Source data are provided in the Source Data file.

67

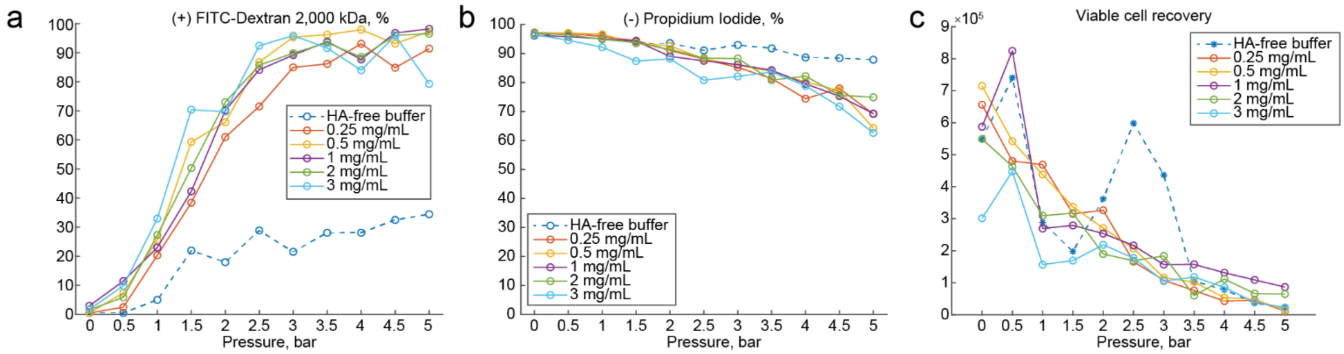

Supplementary Figure 4. Optimization of transfection buffer in the contraction-only device. Jurkat cells were suspended in a transfection buffer consisting of PBS with increasing amounts of hyaluronic acid (HA) and 0.3 mg/mL FITC-Dextran 70 kDa and pumped through device. (a) Delivery efficiency, (b) viability by propidium iodide exclusion, and (c) total number of viable cells recovered were evaluated by imaging flow cytometry about 90 minutes after stretching. A pressure of '0 bar' on the x-axis is used for samples which were incubated with the transfection buffer but not processed through the microfluidic chip. $n = 1$ replicate per condition. Source data are provided in the Source Data file.

68

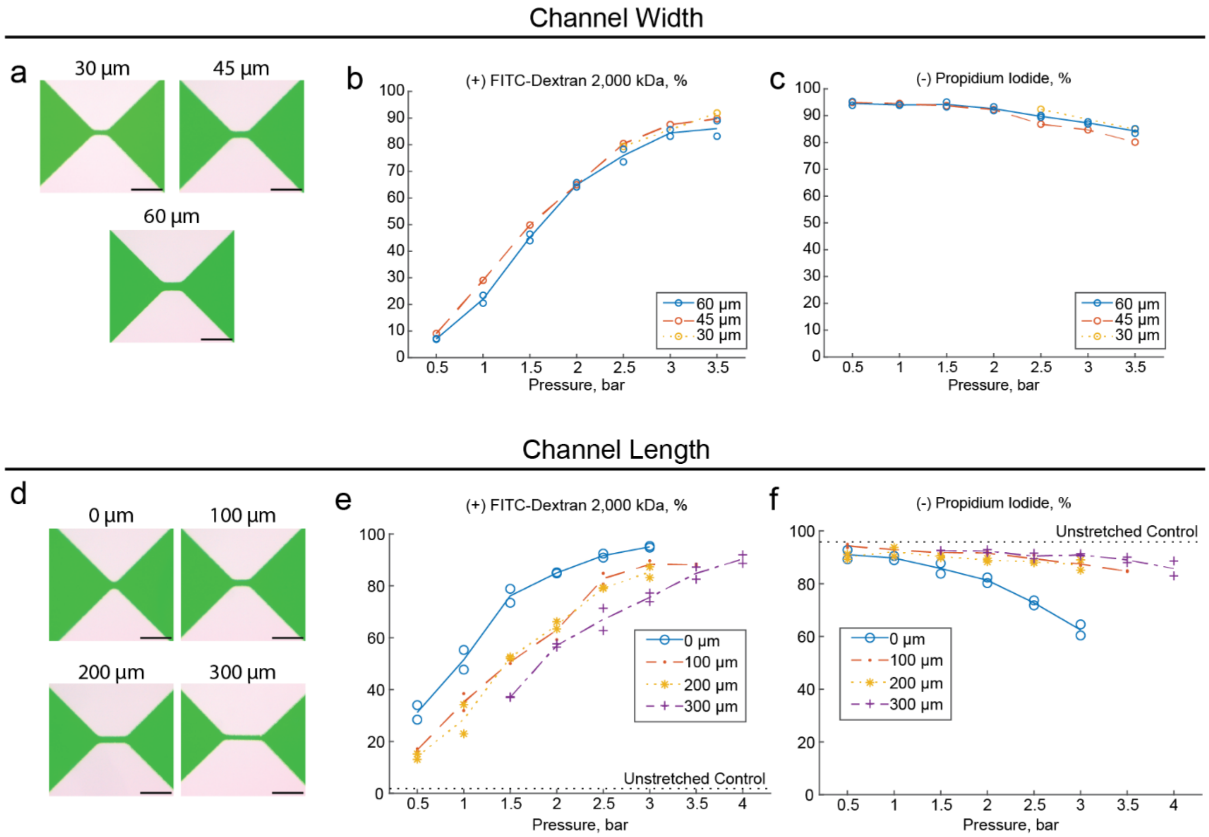

Supplementary Figure 5. Mechanoporation performance for differing channel widths and lengths. Jurkat cells were suspended in a transfection buffer containing PBS with 2 mg/mL hyaluronic acid and 0.3 mg/mL FITC-Dextran 70 kDa and pumped through devices with different channel widths (a-b) and lengths (e-f) at a range of pressures. (a) Images of devices with channel widths 30 µm, 45 µm, or 60 µm wide (length was fixed at 100 µm). (b) Delivery efficiency and (c) viability by propidium iodide exclusion 24 hours after cell stretching. (d) Images of devices with increasing channel lengths (width was fixed at 45 µm). (e) Delivery efficiency and (f) viability by propidium iodide exclusion 24 hours after cell stretching. Lines indicate replicate mean values ($n = 2$ replicates per condition). Scalebars in (a) and (d) indicate 250 µm. Source data are provided in the Source Data file.

69

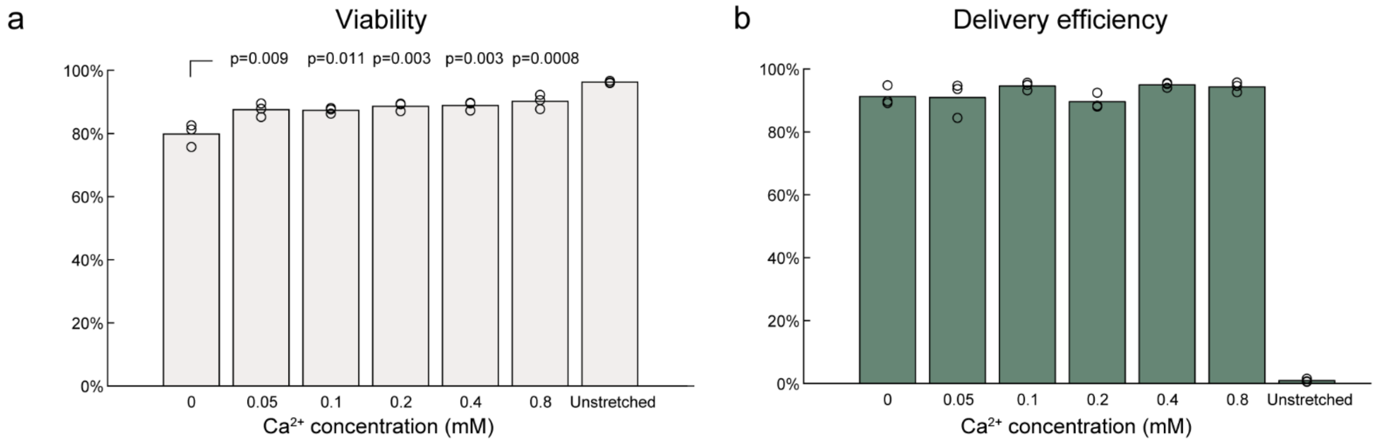

Supplementary Figure 6. Addition of calcium ion improves same-day viability. Jurkat cells were suspended in transfection buffer of PBS with 2 mg/mL hyaluronic acid, 0.2 mg/mL FITC-Dextran 70 kDa, and varying amounts of Ca^{2+}. Suspensions were processed at 3 bar through contraction-only devices 45 μm wide and 100 μm long. (a) Viability for conditions containing calcium were each compared to the no-calcium condition by one-way ANOVA with Tukey's honest significant difference correction, yielding indicated p-values. (b) Corresponding delivery efficiency of 70 kDa FITC-dextran. Bar plots indicate replicate mean values ($n = 3$ replicates for each condition). Source data are provided in the Source Data file.

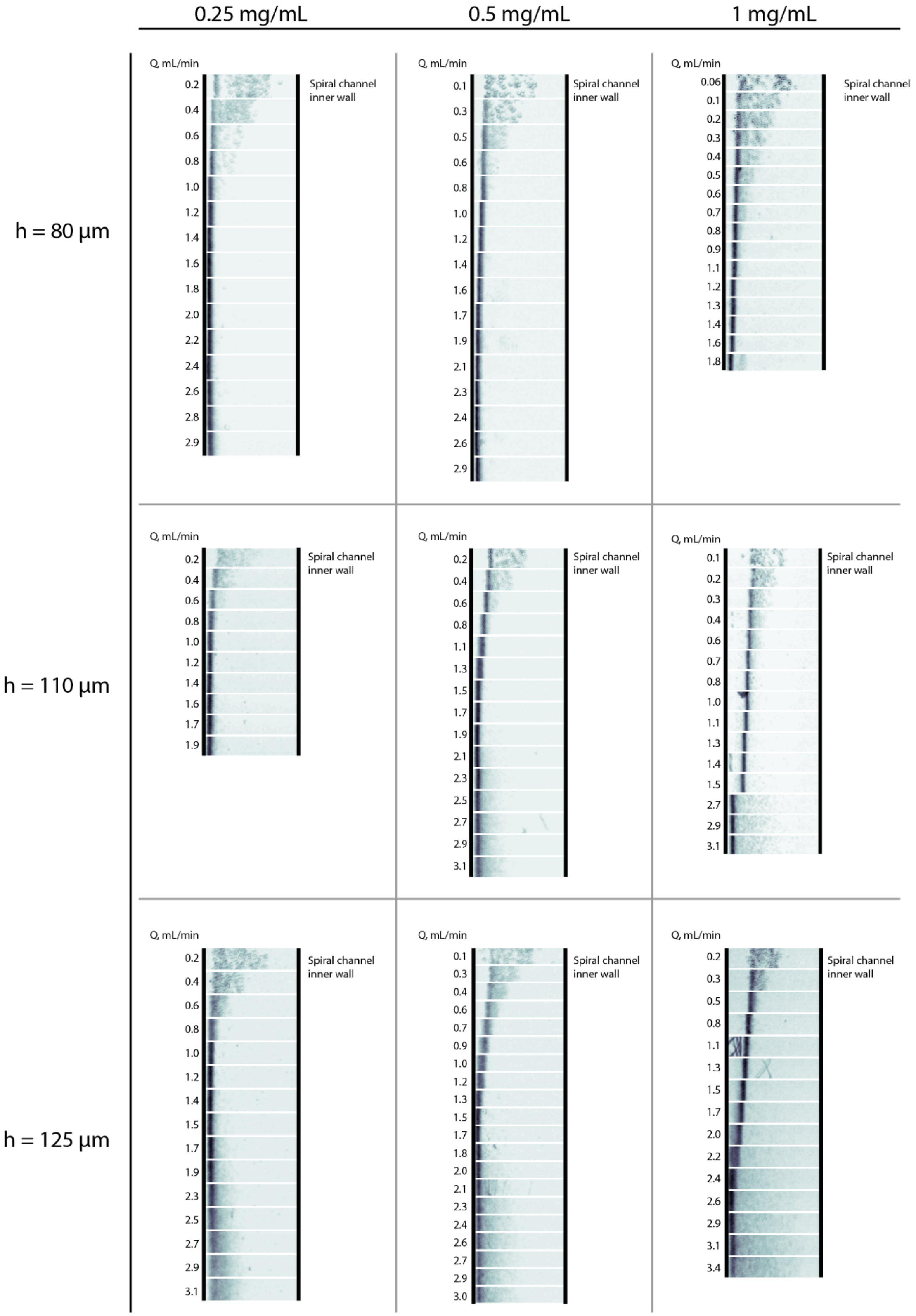

Supplementary Figure 7. Composite time-lapse images of Jurkat cells in 500 µm wide spiral focusing channels, evaluated over a range of flow rates, for each combination of channel height (rows) and hyaluronic acid concentration (columns).

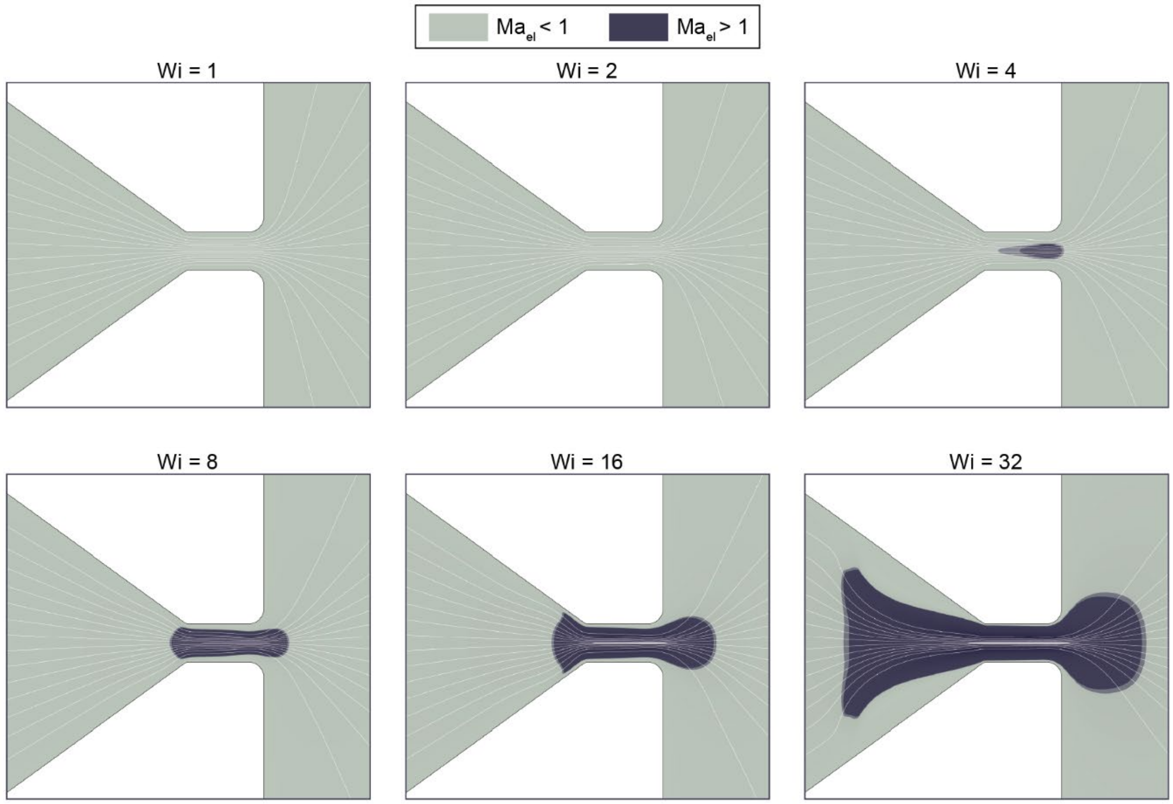

Supplementary Figure 8. Deviations from the laminar flow profile and upstream flow separation are associated with the local viscoelastic Mach number exceeding one in computational simulations. Regions where the local viscoelastic Mach number is either smaller (green) or larger (blue) than one are visualized for increasing Weissenberg numbers across the plane halfway between the channel upper and lower parallel walls. Streamlines show deviations from laminar flow starting at Wi = 4 and upstream separation at Wi = 32, coinciding with local transitions to $Ma_{el} > 1$.

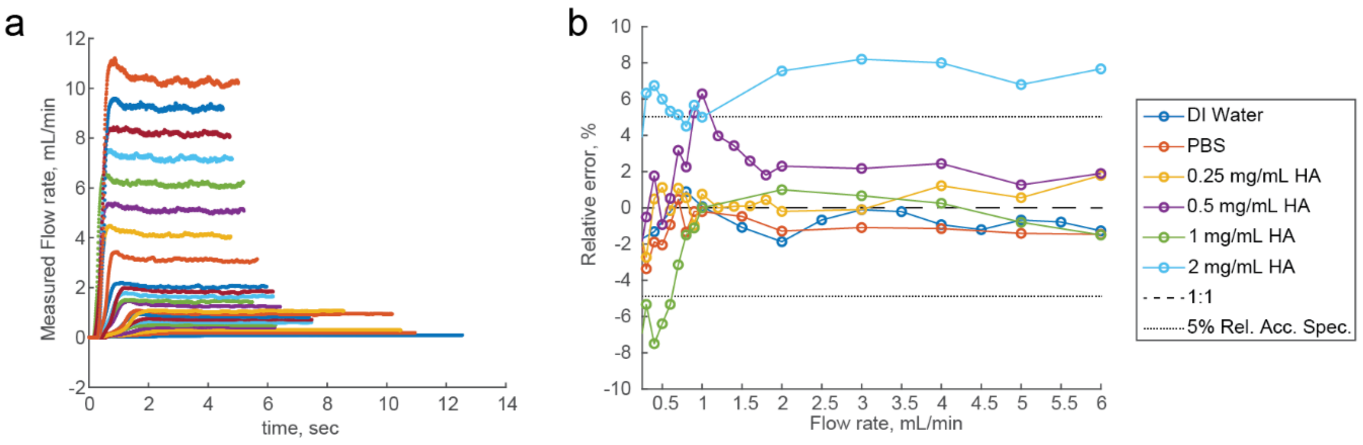

Supplementary Figure 9. Validation of the heat pulse flow meter (Sensirion SLF35-1300F) for solutions of hyaluronic acid (HA). Solutions of 1.6 MDa HA in PBS at concentrations of 0 (none), 0.25 mg/mL, 0.5 mg/mL, 1 mg/mL, and 2 mg/mL. Solutions were driven using a precision syringe pump (Harvard Apparatus) through the flow meter. (a) Representative traces of measured flow rates over time revealed a pumping equilibration time of 2-5 seconds. (b) Tukey mean difference plot of the relative error between measured (flow meter) and prescribed (syringe pump) flow rates. Dotted lines indicate the 5% relative accuracy specification of the flow meter. Lines are provided to guide the eye ($n = 1$ replicate per condition). Source data are provided in the Source Data file.

74

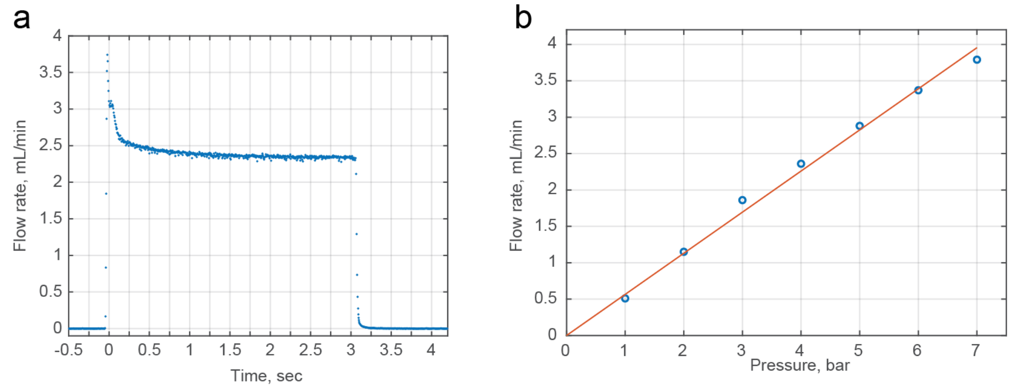

Supplementary Figure 10. Flow rate vs. driving pressure for an 0.5 mg/mL HA solution in the chip with cell focusing channels. (a) Representative time trace of measured flow rate upon opening the pneumatic valve (4 bar). (b) Measured flow rates (Q) at pressures (P) from 1 bar to 7 bar, with linear regression starting at origin ($n = 1$ replicate per condition). Source data are provided in the Source Data file.

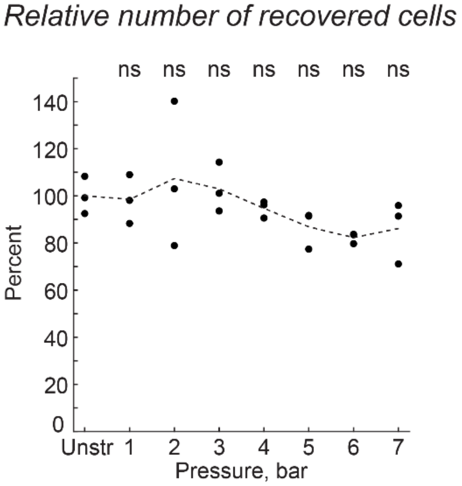

Supplementary Figure 11. Relative number of recovered cells. The number of recovered cells following viscoelastic mechanoporation at a range of operating pressures, relative to the average number of cells in the unstretched control samples ('Unstr') which were not processed through the chip ($n = 3$ replicates per condition). Dotted line indicates sample means. 'ns' indicate means not significantly different ($p<0.05$ criterion) than that of unstretched controls (one-way ANOVA with Tukey's honest significant difference correction). Source data are provided in the Source Data file.

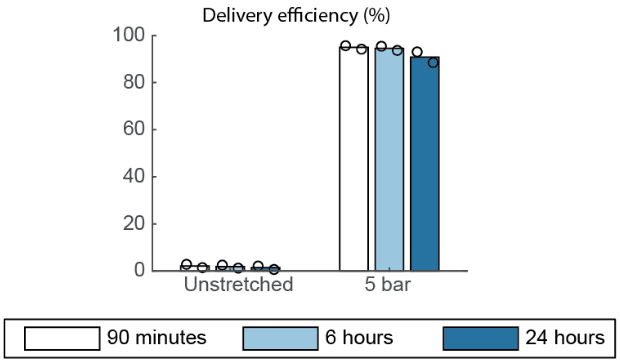

Supplementary Figure 12. FITC-Dextran retention during proliferation. Percent of Jurkat cells staining positive for 70 kDa FITC-Dextran after 90 minutes, 6 hours, and 24 hours post-transfection, as compared to unstretched controls ($n = 2$ replicates per condition). Source data are provided in the Source Data file.

78

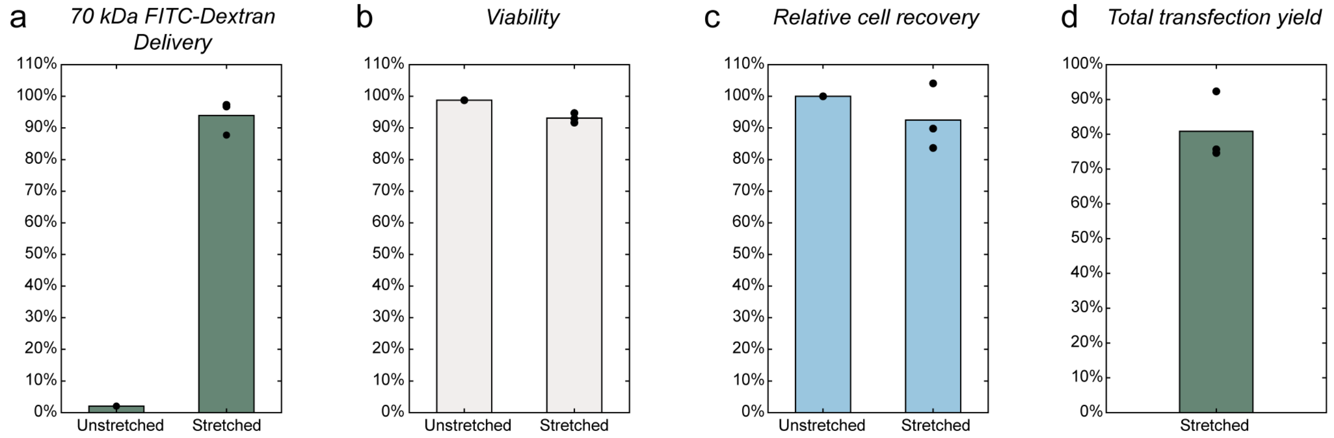

Supplementary Figure 13. Feasibility of large volume transfections. (a) Delivery efficiency of 70 kDa FITC-dextran, (b) viability, (c) number of recovered cells relative to input, and (d) transfection yield for 500 μL samples of 25 million cells, evaluated about 90 minutes after processing. 'Unstretched' indicate cells that were exposed to the delivery solution but not processed through the chip ($n = 1$), while 'Stretched' samples were processed through the chip at 5 bar ($n = 3$ replicates per condition). Source data are provided in the Source Data file.

79

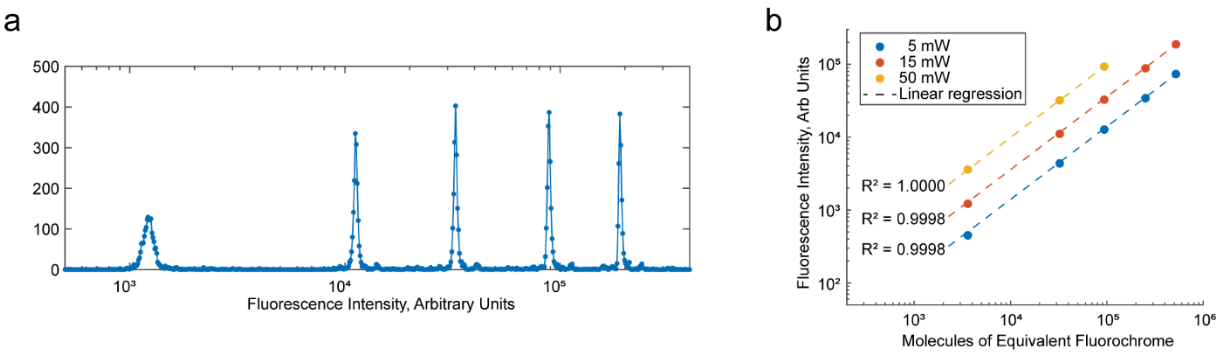

c

	Calibration beads, 2.35e6 beads/mL	FITC-Dextran, 500 ng/mL		Blank buffer
		2,000 kDa	4 kDa	
Average Signal, Arbitary Units	1.6	2.3	1.8	0.26
Molecules of equivalent fluorochrome	17,280 /bead	0.29 /attogram	0.19 /attogram	

Supplementary Figure 14. Flow cytometer calibration for quantitative measurements of dextran delivery. (a) Fluorescence intensity histogram of the calibration bead mixture in the FITC channel of the flow cytometer with an illumination intensity of 15 mW. (b) Calibration bead peak intensities plotted against molecules of equivalent fluorochrome (MEF) for FITC of each bead population, as provided by the manufacturer, at relevant cytometer laser powers. Linear regressions and goodness of fit were calculated on the raw (i.e., not log-transformed) data. (c) Average fluorescence signal measured by the fluorescence plate reader and corresponding molecules of equivalent fluorochrome for the calibration beads and FITC-dextran species. Source data are provided in the Source Data file.

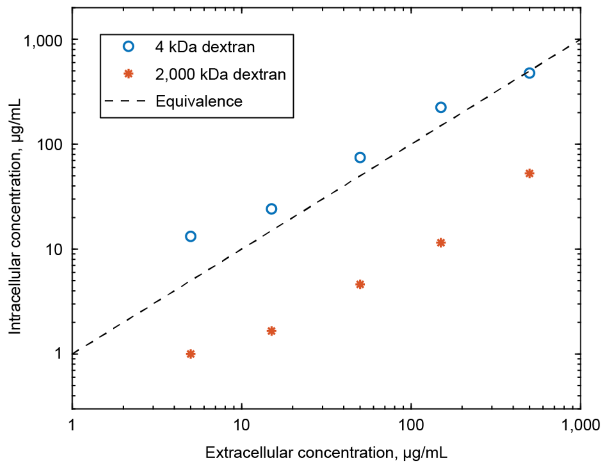

Supplementary Figure 15. Estimated intracellular concentrations of small and large dextrans. Estimates approximate the cell as a sphere with a diameter of 12 µm (e.g., Supplementary Figure 2, $n = 1$ replicate per condition). Source data are provided in the Source Data file.

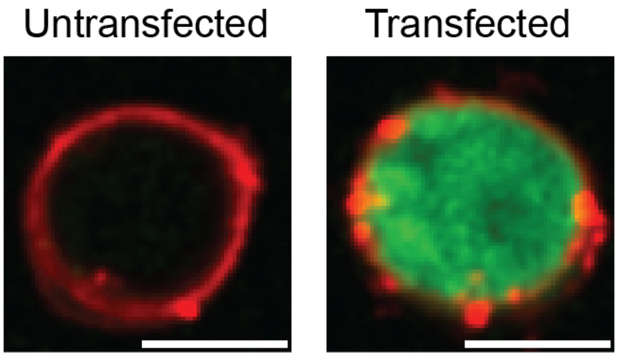

Supplementary Figure 16. 60x Confocal images of FITC-dextran delivered to Jurkat cells. The plasma membrane was covalently labeled (red, Membrite Fix 640) immediately before transfecting 70 kDa FITC-dextran (green) by viscoelastic mechanoporation. Scalebars indicate 10 µm.

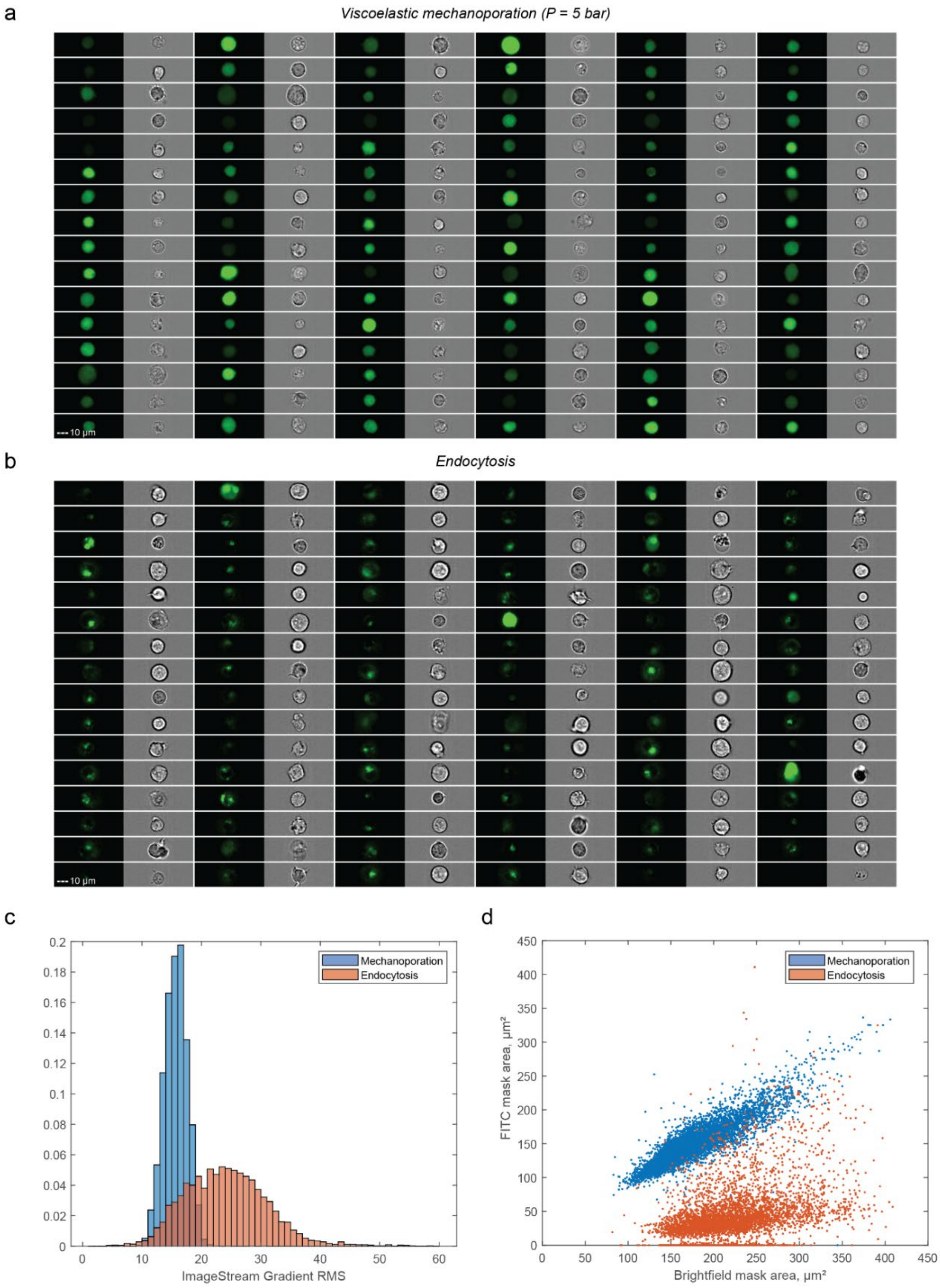

Supplementary Figure 17. Population-level comparisons of 70 kDa FITC-dextran distribution following mechanoporation or passive endocytosis. Representative paired FITC (left, green) and brightfield (right, grayscale) images of Jurkat cells (a) immediately after delivery by viscoelastic mechanoporation or (b) endocytosis. FITC intensities in (a) are about 75x greater than in (b). (c) Histograms of the ImageStream Gradient RMS metric of FITC intensity distribution (lower indicates a smoother image and more uniform distribution). (d) Scatterplots of ImageStream signal-masked area in brightfield versus FITC images (higher FITC mask area indicates FITC is detectable across a larger area within the cell). Source data are provided in the Source Data file.

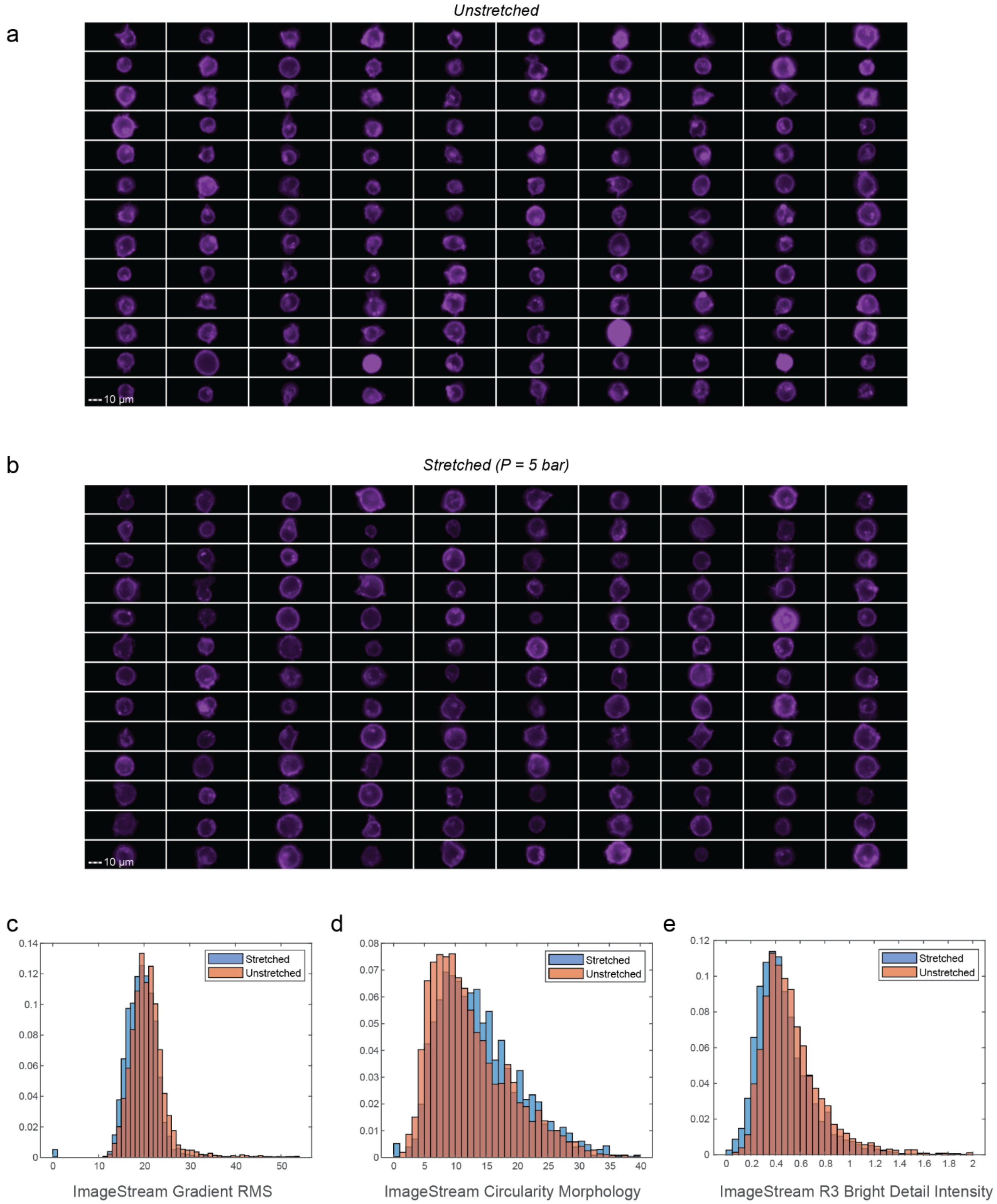

Supplementary Figure 18. Quantitative, population-level comparisons of membrane morphology between stretched and unstretched cells. Jurkat cell plasma membranes were covalently labeled and then imaged (a) directly or (b) about 15 minutes after viscoelastic mechanoporation. Histograms of ImageStream metrics of cell membrane (c) texture, (d) circularity, and (e) brightness of detected puncta in images. Source data are provided in the Source Data file.

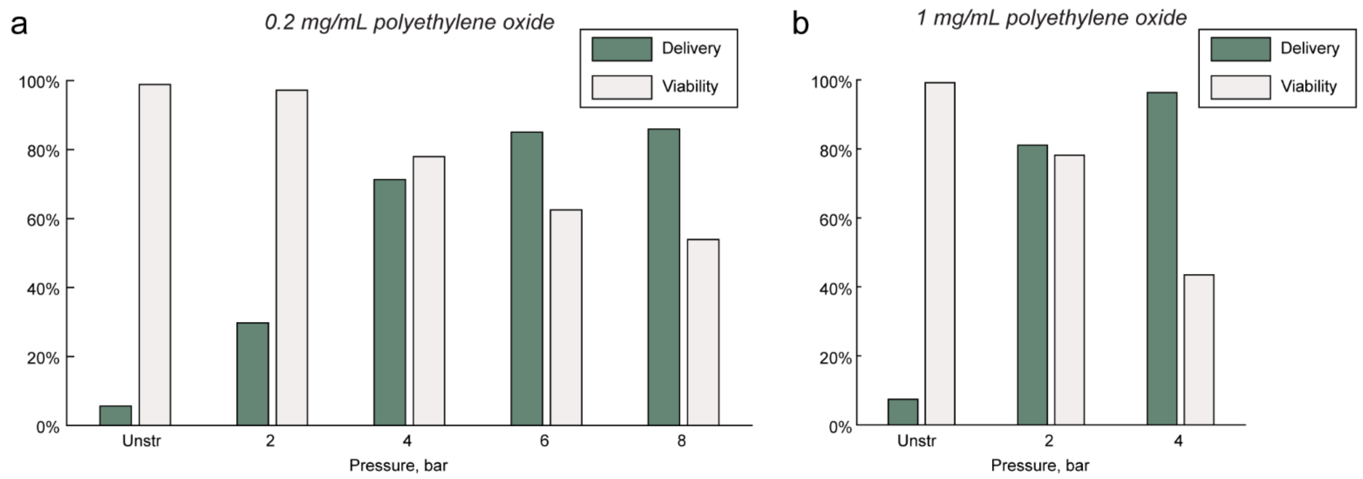

Supplementary Figure 19. Generalization of viscoelastic mechanoporation beyond hyaluronic acid. Viability by propidium iodide exclusion and delivery efficiency of 70 kDa FITC-dextran to Jurkat cells in a PBS solution containing (a) 0.2 mg/mL or (b) 1 mg/mL of 2 MDa polyethylene oxide (PEO, $n = 1$ replicate per condition). Delivery & viability evaluated on the same day as transfection. Source data are provided in the Source Data file.

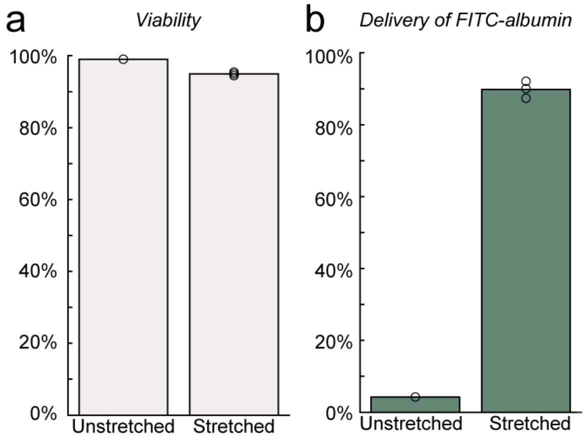

Supplementary Figure 20. Intracellular delivery of protein to Jurkat cells. (a) Viability by propidium iodide exclusion and (b) Delivery efficiency of FITC-albumin evaluated 90 minutes after viscoelastic mechanoporation ($n = 1$ unstretched control sample, $n = 3$ replicate stretched samples). Source data are provided in the Source Data file.

86

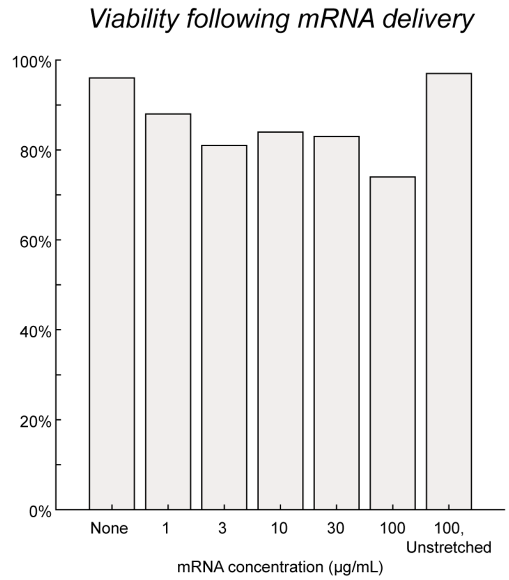

Supplementary Figure 21. Viability of Jurkat cells following mRNA delivery. Viability was assessed by propidium iodide exclusion 24 hours after transfection ($n = 1$ replicate per condition). Source data are provided in the Source Data file.

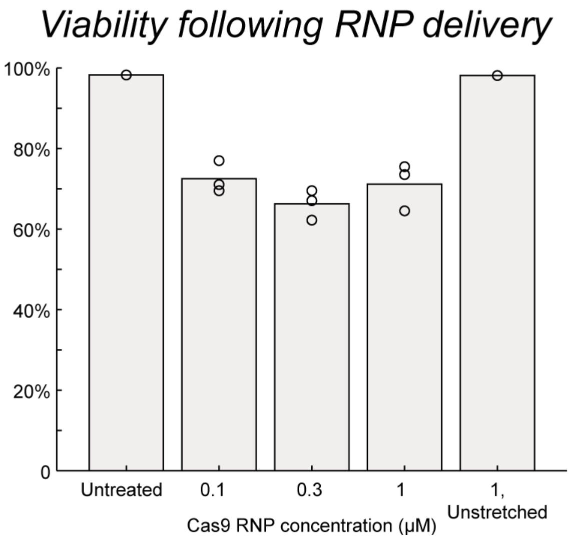

Supplementary Figure 22. Viability of Jurkat cells 2 days after RNP delivery. Viability was assessed by propidium iodide exclusion ($n = 1$ replicate for untreated and unstretched samples, $n = 2$ replicates for each experimental test condition). Source data are provided in the Source Data file.

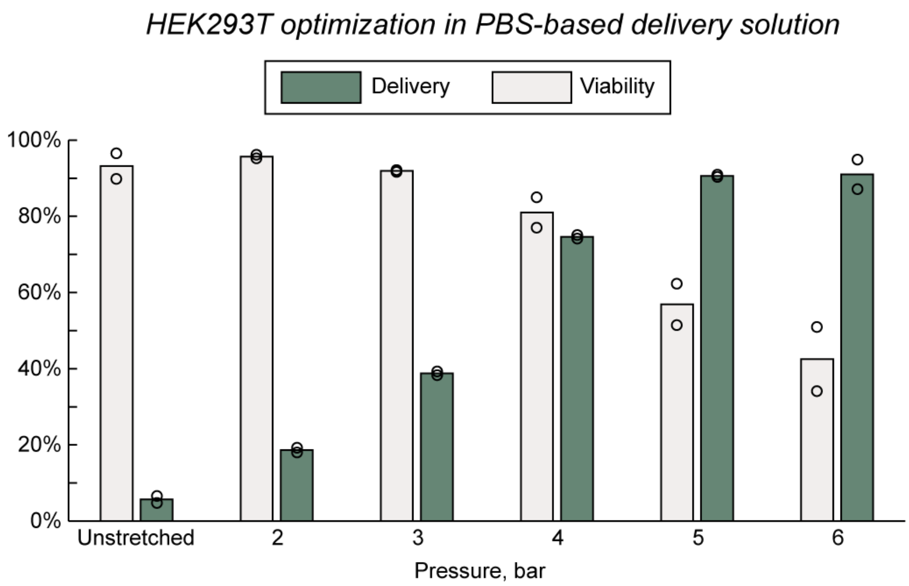

Supplementary Figure 23. Optimization of HEK293T cell transfection. Delivery of 70 kDa FITC-dextran and cell viability measured by propidium iodide exclusion, evaluated 90 minutes after processing through the chip, with PBS-based transfection buffer ($n = 2$ replicates per condition). Source data are provided in the Source Data file.

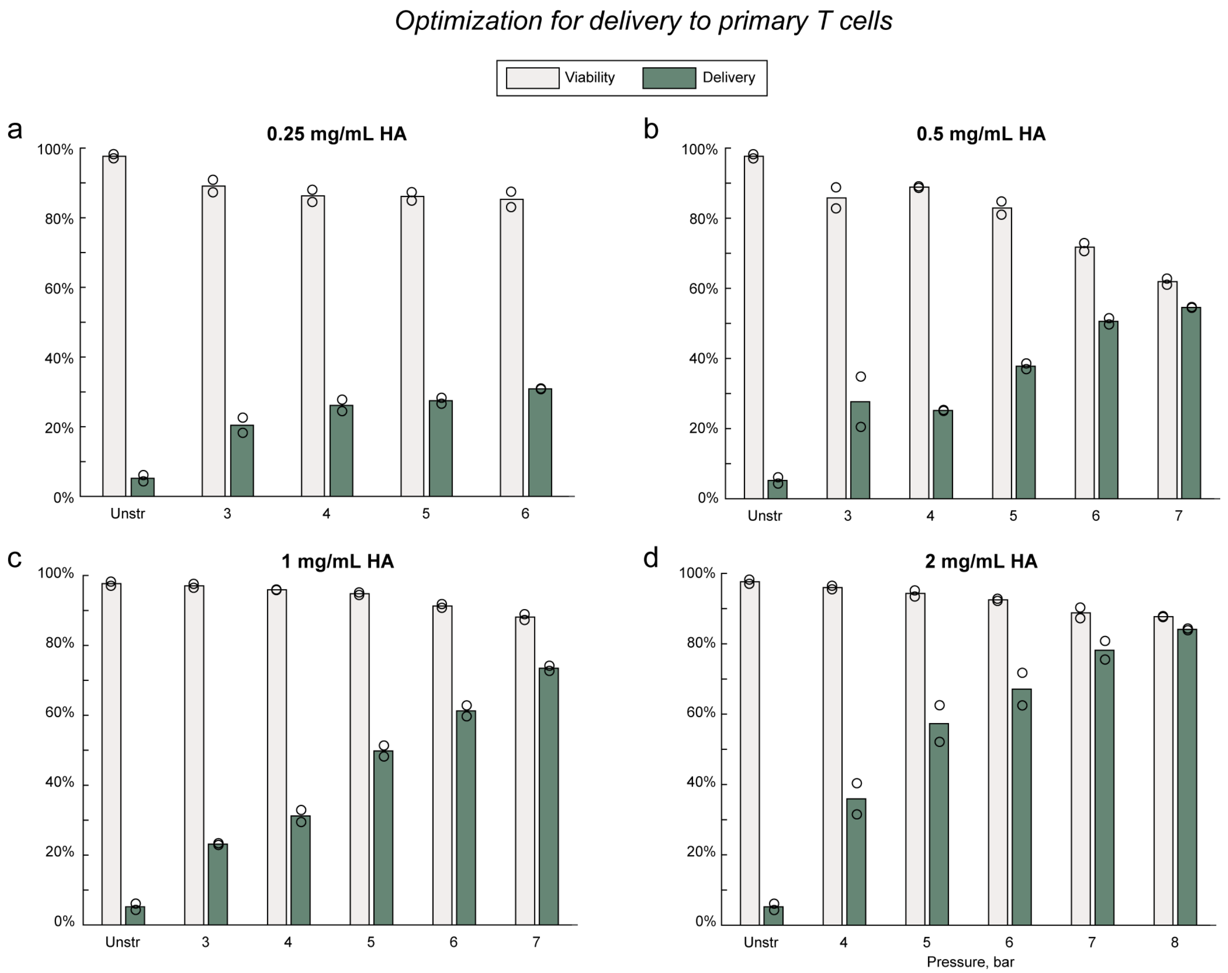

Supplementary Figure 24. Optimization of primary T cell transfection with 70 kDa FITC-dextran. Delivery efficiency and viability were evaluated the same day following transfection with a range of driving pressures for delivery solutions containing (a) 0.25 mg/mL, (b) 0.5 mg/mL, (c) 1 mg/mL, or (d) 2 mg/mL hyaluronic acid (HA). $n = 2$ replicates per condition. Source data are provided in the Source Data file.

91

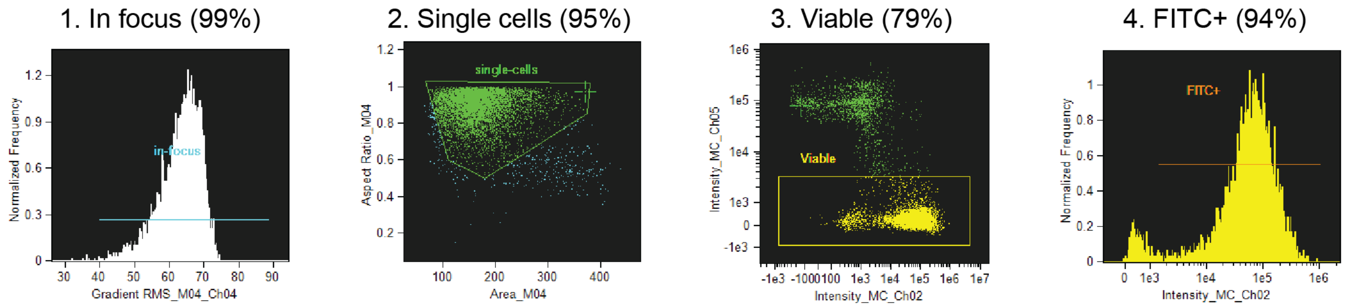

Supplementary Figure 25. Example of ImageStream flow cytometry sequential gating scheme used to assess cell viability and dextran delivery (i.e., sample from Figure 3c-e with operating pressure of 7 bar). Gates were applied sequentially to the population of interest, as numbered and shown from left to right, with the percent of gated objects shown for this example: (1) in-focus images (gradient RMS of brightfield image >40, Channel 4), (2) single cells (based on object size and aspect ratio in brightfield image), viable cells (i.e., propidium iodide negative, Channel 5), FITC+ cells (Channel 2). Source data are provided in the Source Data file.

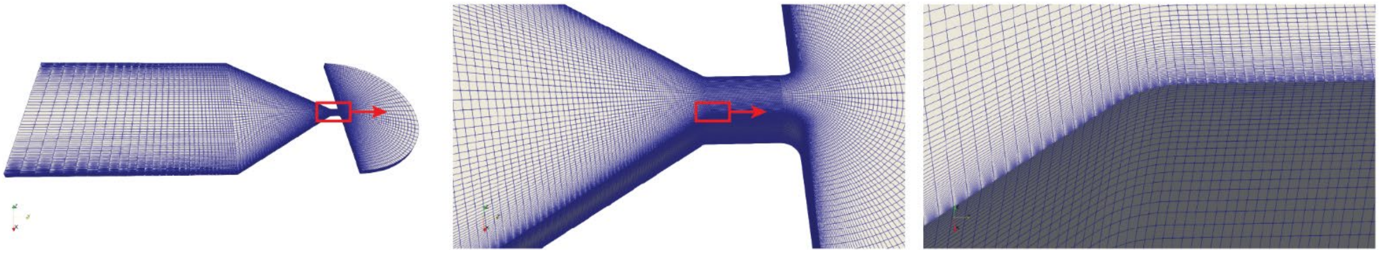

Supplementary Figure 26. Fully swept 3D mesh used for inertio-elastic flow simulations, viewed at three different zoom levels. Surface elements are visualized with opaque gray faces and blue edges. Red rectangles and arrow overlays show the approximate view window of following image on the right. The mesh consists of 2,390,400 hexahedral elements mapped to the geometry with 60 elements across the channel height (i.e., between flat and parallel faces), 120 elements across the width, and 332 along the length (i.e., the direction of flow). The mesh file is available at https://github.com/derinsevenler/viscoelastic-mechanoporation-rheotool-natcomm2023.

Supplementary References

1. Krause, W. E., Bellomo, E. G. & Colby, R. H. Rheology of Sodium Hyaluronate under Physiological Conditions. *Biomacromolecules* **2**, 65–69 (2001).
2. Bingöl, Ö., Lohmann, D., Püschel, K. & Kulicke, W.-M. Characterization and comparison of shear and extensional flow of sodium hyaluronate and human synovial fluid. *Biorheology* **47**, 205–24 (2010).
3. Haward, S. J., Jaishankar, A., Oliveira, M. S. N., Alves, M. A. & McKinley, G. H. Extensional flow of hyaluronic acid solutions in an optimized microfluidic cross-slot device. *Biomicrofluidics* **7**, 044108 (2013).
4. Rodd, L. E., Cooper-White, J. J., Boger, D. V. & McKinley, G. H. Role of the elasticity number in the entry flow of dilute polymer solutions in micro-fabricated contraction geometries. *Journal of Non-Newtonian Fluid Mechanics* **143**, 170–191 (2007).
5. Shi, X. & Christopher, G. F. Growth of viscoelastic instabilities around linear cylinder arrays. *Physics of Fluids* **28**, 124102 (2016).
6. Datta, S. S. *et al.* Perspectives on viscoelastic flow instabilities and elastic turbulence. *Phys. Rev. Fluids* **7**, 080701 (2022).
7. Spiegelberg, S. H., Ables, D. C. & McKinley, G. H. The role of end-effects on measurements of extensional viscosity in filament stretching rheometers. *Journal of Non-Newtonian Fluid Mechanics* **64**, 229–267 (1996).
8. Kim, S. G., Ok, C. M. & Lee, H. S. Steady-state extensional viscosity of a linear polymer solution using a differential pressure extensional rheometer on a chip. *Journal of Rheology* **62**, 1261–1270 (2018).
9. Kwon, C. & Chung, A. J. Highly efficient mRNA delivery with nonlinear microfluidic cell stretching for cellular engineering. *Lab Chip* (2023) doi:10.1039/D2LC01115H.
10. Hur, J. *et al.* Microfluidic Cell Stretching for Highly Effective Gene Delivery into Hard-to-Transfect Primary Cells. *ACS Nano* (2020) doi:10.1021/acsnano.0c05169.
11. Kizer, M. E. *et al.* Hydroporator: a hydrodynamic cell membrane perforator for high-throughput vector-free nanomaterial intracellular delivery and DNA origami biostability evaluation. *Lab Chip* (2019) doi:10.1039/C9LC00041K.

12. Deng, Y. *et al.* Intracellular Delivery of Nanomaterials via an Inertial Microfluidic Cell Hydroporator. *Nano Lett.* **18**, 2705–2710 (2018).

13. Kang, G. *et al.* Intracellular Nanomaterial Delivery via Spiral Hydroporation. *ACS Nano* (2020) doi:10.1021/acsnano.9b07930.

14. Jarrell, J. A. *et al.* Numerical optimization of microfluidic vortex shedding for genome editing T cells with Cas9. *Sci Rep* **11**, 11818 (2021).

15. Jarrell, J. A. *et al.* Intracellular delivery of mRNA to human primary T cells with microfluidic vortex shedding. *Scientific Reports* **9**, 3214 (2019).

16. Ding, X. *et al.* High-throughput nuclear delivery and rapid expression of DNA via mechanical and electrical cell-membrane disruption. *Nature Biomedical Engineering* **1**, 0039 (2017).

17. Lissandrello, C. A. *et al.* High-throughput continuous-flow microfluidic electroporation of mRNA into primary human T cells for applications in cellular therapy manufacturing. *Scientific Reports* **10**, 18045 (2020).

18. Liu, A. *et al.* Microfluidic generation of transient cell volume exchange for convectively driven intracellular delivery of large macromolecules. *Materials Today* **21**, 703–712 (2018).

19. Dixit, H. G. *et al.* Massively-Parallelized, Deterministic Mechanoporation for Intracellular Delivery. *Nano Lett.* **20**, 860–867 (2020).

20. Meacham, J. M., Durvasula, K., Degertekin, F. L. & Fedorov, A. G. Enhanced intracellular delivery via coordinated acoustically driven shear mechanoporation and electrophoretic insertion. *Scientific Reports* **8**, 3727 (2018).
