# Supplementary material for: High throughput intracellular delivery by viscoelastic mechanoporation
Source: Nat Commun. 2024 Jan 2;15:115. doi: 10.1038/s41467-023-44447-w (PMC10762167; doi:10.1038/s41467-023-44447-w)
Supplement: Supplementary file 3 — Reporting Summary [file 41467_2023_44447_MOESM3_ESM.pdf]

nature portfolio

Corresponding author(s): Derin Sevenler & Mehmet Toner

Last updated by author(s): Nov 15, 2023

Reporting Summary

Nature Portfolio wishes to improve the reproducibility of the work that we publish. This form provides structure for consistency and transparency in reporting. For further information on Nature Portfolio policies, see our Editorial Policies and the Editorial Policy Checklist.

Statistics

For all statistical analyses, confirm that the following items are present in the figure legend, table legend, main text, or Methods section.

n/a	Confirmed	
	☒	The exact sample size (*n*) for each experimental group/condition, given as a discrete number and unit of measurement
	☒	A statement on whether measurements were taken from distinct samples or whether the same sample was measured repeatedly
	☒	The statistical test(s) used AND whether they are one- or two-sided<br>*Only common tests should be described solely by name; describe more complex techniques in the Methods section.*
☒		A description of all covariates tested
	☒	A description of any assumptions or corrections, such as tests of normality and adjustment for multiple comparisons
	☒	A full description of the statistical parameters including central tendency (e.g. means) or other basic estimates (e.g. regression coefficient) AND variation (e.g. standard deviation) or associated estimates of uncertainty (e.g. confidence intervals)
	☒	For null hypothesis testing, the test statistic (e.g. *F*, *t*, *r*) with confidence intervals, effect sizes, degrees of freedom and *P* value noted<br>*Give P values as exact values whenever suitable.*
☒		For Bayesian analysis, information on the choice of priors and Markov chain Monte Carlo settings
☒		For hierarchical and complex designs, identification of the appropriate level for tests and full reporting of outcomes
☒		Estimates of effect sizes (e.g. Cohen's *d*, Pearson's *r*), indicating how they were calculated

Our web collection on statistics for biologists contains articles on many of the points above.

Software and code

Policy information about availability of computer code

Data collection	Rheotool (https://github.com/fppimenta/rheoTool) and OpenFOAM v9 (https://openfoam.org/) were used for computational fluid dynamics simulations.
Data analysis	Plotting and statistical tests were performed with MATLAB v2019a. Flow cytometry analysis was performed with Imagestream IDEAS version 6.2.

For manuscripts utilizing custom algorithms or software that are central to the research but not yet described in published literature, software must be made available to editors and reviewers. We strongly encourage code deposition in a community repository (e.g. GitHub). See the Nature Portfolio guidelines for submitting code & software for further information.

Data

Policy information about availability of data

All manuscripts must include a data availability statement. This statement should provide the following information, where applicable:

- Accession codes, unique identifiers, or web links for publicly available datasets
- A description of any restrictions on data availability
- For clinical datasets or third party data, please ensure that the statement adheres to our policy

Source data are provided with this paper. Raw videos, raw flow cytometry data, and raw numerical simulation solutions are available from the corresponding author upon reasonable request.

Research involving human participants, their data, or biological material

Policy information about studies with human participants or human data. See also policy information about sex, gender (identity/presentation), and sexual orientation and race, ethnicity and racism.

Reporting on sex and gender	This information was not collected and sex/gender was not discussed in this manuscript.
Reporting on race, ethnicity, or other socially relevant groupings	No information related to race, ethnicity, or other socially relevant groupings was collected or discussed in this manuscript.
Population characteristics	This information was not collected and population characteristics were not discussed in this manuscript.
Recruitment	No donor or patient recruitment was undertaken.
Ethics oversight	No explicit ethics oversight was performed for this study.

Note that full information on the approval of the study protocol must also be provided in the manuscript.

Field-specific reporting

Please select the one below that is the best fit for your research. If you are not sure, read the appropriate sections before making your selection.

☒ Life sciences ☐ Behavioural & social sciences ☐ Ecological, evolutionary & environmental sciences

For a reference copy of the document with all sections, see nature.com/documents/nr-reporting-summary-flat.pdf

Life sciences study design

All studies must disclose on these points even when the disclosure is negative.

Sample size	For intracellular delivery studies, n=1 or n=2 replicates were initially used (as indicated) for preliminary screening across the potentially vast design parameter space (flow rate, geometry height, width, length, hyaluronic acid concentration, biomolecule concentration, buffer composition). For characterization experiments, n=3 replicates per condition were used to identify potential outliers (ultimately no outliers were excluded). No statistical tests were used to determine the required sample size.
Data exclusions	No data were excluded from analysis.
Replication	All experiments were performed independently. The number of replicates of each experiment are included in the manuscript. The authors have reproduced the experimental findings with consistent results.
Randomization	For each test, cells from a single mixed population were randomly allocated to the experimental conditions.
Blinding	Blinding was not always possible since one scientist designed, performed, and analyzed all experiments.

Reporting for specific materials, systems and methods

We require information from authors about some types of materials, experimental systems and methods used in many studies. Here, indicate whether each material, system or method listed is relevant to your study. If you are not sure if a list item applies to your research, read the appropriate section before selecting a response.

Materials & experimental systems

n/a	Involved in the study	
☐	☒	Antibodies
☐	☒	Eukaryotic cell lines
☒	☐	Palaeontology and archaeology
☒	☐	Animals and other organisms
☒	☐	Clinical data
☒	☐	Dual use research of concern
☒	☐	Plants

Methods

n/a	Involved in the study	
☒	☐	ChIP-seq
☐	☒	Flow cytometry
☒	☐	MRI-based neuroimaging

Antibodies

Antibodies used	FITC anti-human TCR α/β Antibody, Clone IP26
Validation	https://www.biolegend.com/en-us/products/fitc-anti-human-tcr-alpha-beta-antibody-772

From vendor website: "Each lot of this antibody is quality control tested by immunofluorescent staining with flow cytometric analysis."

Eukaryotic cell lines

Policy information about [cell lines and Sex and Gender in Research]

Cell line source(s)	Jurkat E6-1, ATCC (TIB-152); HEK293T, ATCC (293T/17)
Authentication	No independent authentication was performed.
Mycoplasma contamination	No testing for mycoplasma was performed.
Commonly misidentified lines (See ICLAC register)	No misidentified cell lines were used.

Plants

Seed stocks	*Report on the source of all seed stocks or other plant material used. If applicable, state the seed stock centre and catalogue number. If plant specimens were collected from the field, describe the collection location, date and sampling procedures.*
Novel plant genotypes	*Describe the methods by which all novel plant genotypes were produced. This includes those generated by transgenic approaches, gene editing, chemical/radiation-based mutagenesis and hybridization. For transgenic lines, describe the transformation method, the number of independent lines analyzed and the generation upon which experiments were performed. For gene-edited lines, describe the editor used, the endogenous sequence targeted for editing, the targeting guide RNA sequence (if applicable) and how the editor was applied.*
Authentication	*Describe any authentication procedures for each seed stock used or novel genotype generated. Describe any experiments used to assess the effect of a mutation and, where applicable, how potential secondary effects (e.g. second site T-DNA insertions, mosiacism, off-target gene editing) were examined.*

Flow Cytometry

Plots

Confirm that:

- [x] The axis labels state the marker and fluorochrome used (e.g. CD4-FITC).
- [x] The axis scales are clearly visible. Include numbers along axes only for bottom left plot of group (a 'group' is an analysis of identical markers).
- [x] All plots are contour plots with outliers or pseudocolor plots.
- [x] A numerical value for number of cells or percentage (with statistics) is provided.

Methodology

Sample preparation	Cells were washed by centrifugation, resuspended in 50 uL of phosphate buffered saline with 2 ug/mL of propidium iodide, and incubated at room temperature for 10-30 minutes before measurement.
Instrument	Amnis ImageStream Mark II
Software	Imagestream IDEAS version 6.2
Cell population abundance	All input cells were analyzed (the cells of interest represented 100% of input sample).
Gating strategy	The default ImageStream gating approach was used, specifically: 1. Gating of in-focus images by image gradient RMS (~99% of images); 2. Gating of single cells yb feature size and aspect ratio (~90% or more of images); 3. Gating of viable cells to determine population viability. For FITC-dextran delivery experiments, another sequential gate was used on the FITC-channel

- [x] Tick this box to confirm that a figure exemplifying the gating strategy is provided in the Supplementary Information.
